# Supplementary material for: Wound-Healing Effect of Antheraea pernyi Epidermal Growth Factor
Source: Insects. 2022 Oct 24;13(11):975. doi: 10.3390/insects13110975 (PMC9695916; doi:10.3390/insects13110975)
Supplement: Supplementary file 1 [file insects-13-00975-s001.zip › insects-1913509-supplementary.pdf]

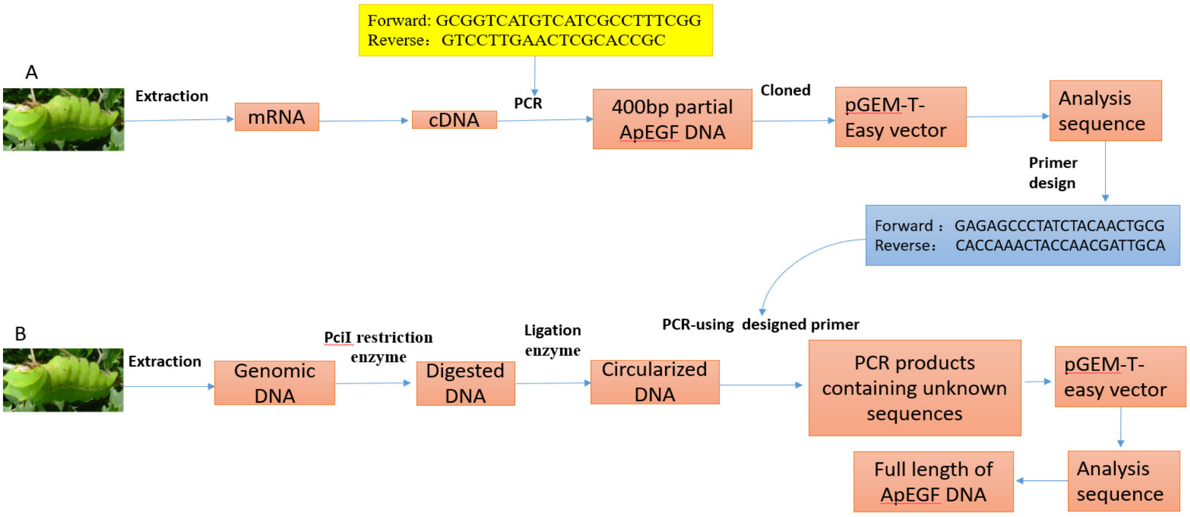

Supplement Figure S1. Diagram that shows the process of obtaining full-length ApEGF gene sequence by inverse PCR.

A) The process of obtaining a partial ApEGF gene sequence by PCR.

B) The process of inverse PCR.

The yellow box indicate the primer sequences were designed based on the *Bombyx mori* pro-epidermal growth factor gene. The blue box indicate the primer sequences were designed based on obtained partial gene sequences of ApEGF gene.

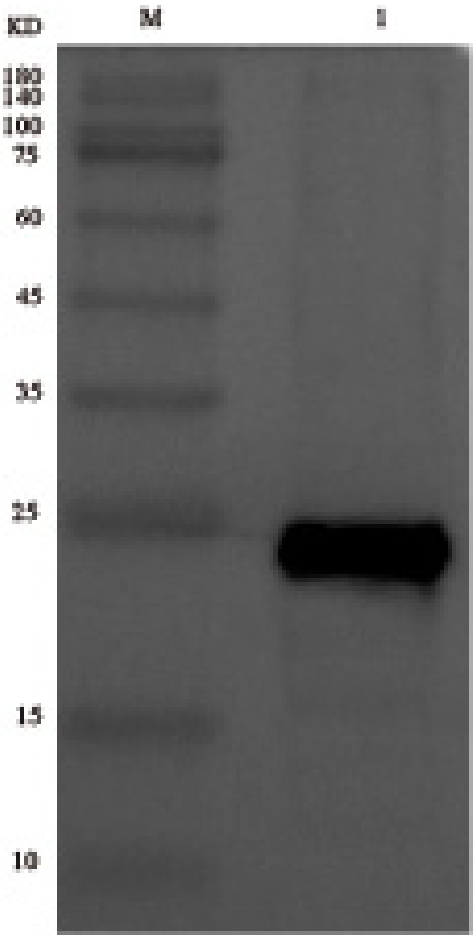

Supplement Figure S2. Western blot analysis of purified cut-off ApEGF with anti-His-tag. Lane M: marker; Lane 1: purified protein

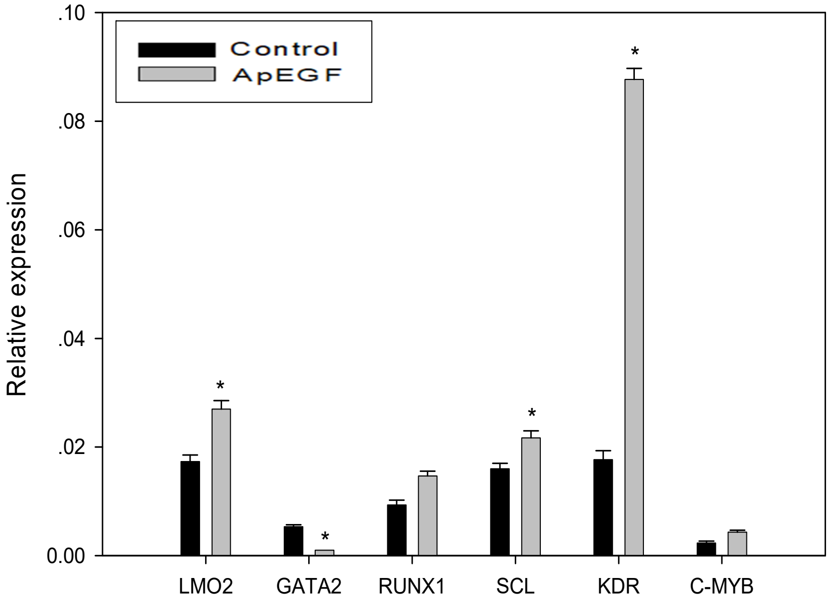

Supplement Figure S3. Effects of truncated ApEGF on the mRNA expression of LMO2, GATA2, RUNX1, SCL, KDR, and C-MYB in HaCaT cells. *, $P<0.05$, significant versus the control.
